# Use of high-dose steroid therapy: addition of anakinra in the treatment of severe COVID-19

**DOI:** 10.1590/1806-9282.20230671

**Published:** 2024-03-15

**Authors:** Kadir Gorkem Guclu, Ceyda Geyiktepe-Guclu, Osman Faruk Bayramlar, Gulsah Tuncer, Mehtap Aydin

**Affiliations:** 1 Umraniye Training and Research Hospital, Department of Infectious Diseases and Clinical Microbiology – İstanbul, Turkey.; 2 Haseki Training and Research Hospital, Department of Infectious Diseases and Clinical Microbiology – İstanbul, Turkey.; 3 Turkish Ministry of Health - Istanbul Health Directorate, Bakırköy District Health Directorate – İstanbul, Turkey.; 4 Bilecik Training and Research Hospital, Department of Infectious Diseases and Clinical Microbiology – Bilecik, Turkey.

**Keywords:** COVID-19, Anakinra, Steroid, Cytokine storm

## Abstract

**OBJECTIVE:**

The aim of this study was to compare the clinical effects of the addition of anakinra to high-dose steroid therapy in COVID-19 patients with macrophage activation syndrome.

**METHODS:**

This was a single-center retrospective study conducted in Ümraniye Training and Research Hospital between March 11, 2020, and April 28, 2021. Patients receiving only high-dose steroid or anakinra+steroid were enrolled. The first day of anakinra was considered as day 0. Laboratory values and oxygen requirements were followed up for 7 days. Patients were divided into two groups: 66 patients in the high-dose steroid group and 67 patients in the anakinra+steroid group. The primary outcome was 28-day mortality.

**RESULTS:**

After treatment, a significant decrease in ferritin levels was detected only in the anakinra+steroid group (p=0.001). In both groups, there were significant changes in lymphocytes, C-reactive protein, lactate dehydrogenase, and fibrinogen levels during the 7-day follow-up. Changes in oxygen status according to the World Health Organization clinical scale on day 3 and day 7 between high-dose steroid and anakinra+steroid groups were similar (p=0.976). Complications were higher in the anakinra+steroid group than in the steroid group (26% vs. 12%, p=0.03). The rates of 28-day mortality were 57% in the anakinra+steroid group and 42% in the high-dose steroid group (p=0.48). In multivariate regression, anakinra did not affect 28-day mortality (p=0.67).

**CONCLUSION:**

The addition of anakinra to steroid treatment resulted in a significant decrease in biochemical parameters. However, no significant difference was observed in the oxygen status between the groups. The addition of anakinra to steroid treatment did not decrease mortality. Clinicians should be aware of the complications of anti-inflammatory therapies.

## INTRODUCTION

The global spread of COVID-19 has affected nearly every country, resulting in a significant number of fatalities, with approximately 6.95 million deaths reported worldwide^1^. Certain laboratory parameters, such as C-reactive protein (CRP), lymphopenia, increased ferritin levels, and thrombocytopenia, have been identified as indicators of poor prognosis in COVID-19 cases^2^. The progression of COVID-19 is often accompanied by an upsurge in proinflammatory endogenous cytokines, including interleukin-1 (IL-1), interleukin-6 (IL-6), and tumor necrosis factor-alpha (TNF-alpha). This situation can lead to MAS, both of which have been linked to severe morbidity and mortality^3^. Notably, studies suggest that anakinra, an IL-1 receptor antagonist, may serve as a potential treatment option for cytokine storms and MAS in COVID-19^4^. This study was conducted to evaluate the clinical outcomes of adding anakinra to high-dose steroid therapy in patients diagnosed with MAS based on clinical and laboratory findings. Our study aims to compare the use of anakinra with high-dose steroid to the use of only high-dose steroid, with an objective of determining any clinical benefits.

## METHODS

### Study design

This was a single-center retrospective study. We included patients who were admitted to the Hospital between March 11, 2020 and April 28, 2021 for COVID-19 management and follow-up. MAS treatment was given in accordance with the COVID-19 guidelines of the Ministry of Health. These recommendations include only high-dose steroid or anakinra with high-dose steroid. According to the guidelines, anakinra was administered intravenously at a maximum dose of 1200 mg/day. The total dose was divided into two or three doses and administered intravenously at equal time intervals. Patients with SpO_2_ <93% or PaO_2_/FiO_2_ <300, CRP >50 mg/L, and ferritin >600 ng/mL or IL-6 >6 ng/μg were considered for MAS treatment. On the day of MAS evaluation, both patient groups were given high-dose steroid. All patients were given 250 mg of methylprednisolone for 3 days. Subsequent doses were reduced according to the clinical situation. Some patients had anakinra with 250 mg of methylprednisolone^5^. Anakinra was added to the treatment of patients who did not have clinical improvement, according to the Ministry of Health guidelines. After the development of MAS, laboratory values and oxygen status for 7 days were retrospectively collected. In addition, both groups were underlying chronic diseases, and clinical and laboratory findings during admission and CT screening results were retrospectively collected. According to the COVID-19 guidelines, at least 50% involvement in unilateral or bilateral was determined as severe CT findings.

These patients were divided into two groups: the first group comprised patients receiving high-dose steroid (steroid group), while the second group comprised patients receiving anakinra in addition to high-dose steroid (anakinra+steroid group). The day of addition of anakinra was considered as day 0 in patients who were added to the anakinra group. Laboratory values and oxygen requirements were compared for 7 days (days 0, 1, 3, 5, and 7). A total of 66 patients were included in the steroid group, and 67 patients were included in the anakinra+steroid group. The primary outcome was 28-day mortality. The secondary outcomes were changes in laboratory values and the WHO's COVID-19 clinical scale. The 28-day mortality rates and intensive follow-up were examined. Patients were grouped according to their oxygen status, as suggested by the WHO's COVID-19 clinical scale^6^.

### Study population

In our study, patients with laboratory-confirmed COVID-19 who developed MAS with clinical and laboratory findings were included. The inclusion criteria for this study were as follows: older than 18 years, patients with developing MAS, and laboratory-confirmed COVID-19. Laboratory-confirmed COVID-19 was defined as patients with a positive SARS-CoV-2 test at least once in oropharyngeal and nasopharyngeal swab samples. The exclusion criteria for this study were as follows: HIV -positive patients, patients with COVID-19 vaccines, and patients with viral hepatitis, active tuberculosis, acute respiratory distress syndrome (ARDS), and neutropenia and uncontrolled diabetes. In addition, patients who died within the first 3 days after steroid or anakinra administration or patients in need of intensive care during admission were excluded. Pregnant patients and patients ≥6 points on the clinical scale of the WHO were excluded^6^.

### Statistical analysis

The SPSS 20 Windows package program (IBM Corp., Armonk, NY, USA) was utilized for data analysis. Key variables included 28-day mortality, the impact of anakinra supplementation on oxygen demand, and laboratory values. Various factors such as sex, age, comorbidities, laboratory values, CT findings, and oxygen status were compared. Categorical variables were presented as numbers and percentages, while continuous variables were expressed as mean±SD or median with interquartile range (IQR). The chi-square test was used to compare categorical variables, while Student's t-test or Mann-Whitney U test was employed for numerical variables depending on their distribution. Statistical significance was set at p<0.05. Variables that exhibited a significant effect on 28-day mortality were included in the univariate logistic regression analysis. In the multivariate analysis, parameters that were statistically significant in the univariate analysis and had an odds ratio of ≥1.01 were considered. The obtained results were assessed with a 95% confidence interval.

## RESULTS

Of the included patients, 62.4% were males and 37.6% were females, with a mean age of 59±14 years. The patient population was divided into two groups: 67 patients in the anakinra+steroid group and 66 patients in the steroid group. The distribution of age, gender, and chronic disease was similar between the two groups (p=0.43) (Table 1). None of the patients had received any COVID-19 vaccine. Severe CT findings were observed in 62.3% of patients in the anakinra+steroid group and 37.7% in the high-dose steroid group (p=0.03). At admission, oxygen requirement, duration of symptoms, fever, pulse rate, and respiratory rate were evaluated, and no significant differences were observed between the two groups (Table 1). At admission, laboratory parameters such as leukocyte count, lymphocyte count, platelet count, aspartate aminotransferase (AST), lactate dehydrogenase (LDH), creatine kinase (CK), C-reactive protein (CRP), D-dimer, and fibrinogen levels were similar in both groups.

**Table 1:** Demographic data of patients, comorbidities, tomographic findings, and vital signs.

Characteristics		Steroid (n=66)	Anakinra+steroid (n=67)	p-value
Age (mean±SD)		60±14	57±14	0.38
Sex				
	Male	39 (47)	44 (53)	0.43
	Female	27 (54)	23 (46)	
Comorbidities		48 (53)	41 (46)	
	Chronic lung disease	10 (59)	7 (41)	0.44
	Diabetes	20 (45)	24 (55)	0.50
	Hypertension	30 (53)	27 (47)	0.55
	Cardiovascular diseases	9 (47)	10 (53)	0.83
	Heart failure	3 (42)	4 (57)	1.00
	Central nervous system diseases	2 (50)	2 (50)	1.00
	Cancer	3 (37.5)	5 (62.5)	0.72
	Chronic kidney disease	4 (57)	3 (43)	0.72
	Rheumatic diseases	0 (0)	2 (100)	0.50
Tomographic findings				
	Mild	46	34 (42.5)	**0.03**
	Severe	(57.5) 20 (38)	33 (62)	
Fever (°C) (mean±SD)		36.9±0.7	36.8±0.6	0.30
Respiratory rate/minute (mean±SD)		24±4	23±4	0.28
Pulse/minute (mean±SD)		88±15.4	90±15.2	0.66
Complaint period (day) (mean±SD)		6±3	7±4	0.58
Admission saturation (%) (mean±SD)		88±5	88±6	0.10

Bold indicates statistically significant p-value.

Moreover, mean ferritin levels were higher in patients receiving anakinra+steroid compared to those receiving only steroid at admission (1136±1323 ng/mL vs. 634±574 ng/mL, p=0.03). ALT levels were higher at admission in the anakinra group (47±46 IU/L vs. 31±21 IU/L, p=0.03). When anti-inflammatory therapy started, leukocyte, thrombocyte, ALT, LDH, and ferritin levels were higher in the anakinra+steroid group. CRP levels were higher in the steroid group (p=0.19). There was no difference in fibrinogen, IL-6, D-dimer, CK, and AST values. The mean IL-6 levels were found to be 41.8±36 pg/mL in the anakinra+steroid group and 27.8±27.5 pg/mL in the steroid group (p=0.21). Intensive care admission was higher in the anakinra+steroid group (68% vs. 32%, p=0.09).

Anakinra was started on the day 11 of symptom onset, and steroids were started on day 9. The mean procalcitonin values measured on the day of treatment were <0.25 ng/mL, and no significant difference was found between the two groups. In both groups, there was no significant change in leukocyte, ALT, CK, or D-dimer levels for 7 days. The decrease in ferritin levels was significant only in the anakinra+steroid group (p=0.001). LDH decrease was observed in both groups (Table 2). The LDH decrease was more significant in the anakinra+steroid group (p=0.001 vs. p=0.005).

**Table 2:** Laboratory findings on days 1, 3, 5, and 7.

Parameters		Anakinra+steroid		p	Steroid		p
		Mean±SD	Median (IQR)		Mean±SD	Median (IQR)	
Platelet count/mm^3^	Day 1	329±142	316 (236–384)	**0.03**	267±92	270 (198–306)	**0.01**
	Day 3	349±126	330 (264–401)		306±119	306 (235–375)	
	Day 5	358±131	351 (278–411)		336±137	327 (267–402)	
	Day 7	341±114	329 (274–395)		316±132	309 (216–370)	
Lymphocyte count/mm^3^	Day 1	774±489	670 (500–890)	**0.001**	792±420	710 (500–1000)	**0.001**
	Day 3	947±669	755 (540–1200)		953±575	815 (525–1325)	
	Day 5	1052±785	920 (530–1280)		1112±706	990 (450–1530)	
	Day 7	1300±1011	1010 (630–1610)		1144±860	1100 (435–1410)	
AST, IU/L	Day 1	40±28	32 (23–43)	**0.03**	43±60	28 (21–49)	**0.08**
	Day 3	39±21	34 (22–52)		44±52	32 (21–44)	
	Day 5	37±21	34 (22–45)		32±17	27 (20–39)	
	Day 7	31±19	24 (19–33)		30±18	23 (19–35)	
	Day 3	69±46	61 (31–98)		58±63	40 (24–76)	
	Day 5	80±58	63 (34–106)		57±47	45 (26–80)	
	Day 7	74±61	49 (29–114)		74±84	51 (29–86)	
LDH, IU/L	Day 1	458±214	418 (310–568)	**0.001**	339±100	326 (286–390)	**0.005**
	Day 3	461±220	401 (333–558)		363±119	339 (276–421)	
	Day 5	400±186	357 (281–485)		313±132	293 (243–349)	
	Day 7	382±234	329 (259–429)		323±118	320 (264–358)	
Ferritin, ng/mL	Day 1	1361±1532	766 (432–2100)	**0.001**	746±665	572 (267–982)	0.40
	Day 3	1064±1000	781 (398–1411)		751±763	548 (310–928)	
	Day 5	925±837	700 (304–1213)		682±654	500 (312–814)	
	Day 7	761±614	583 (328–992)		697±564	478 (360–983)	
CRP, mg/L	Day 1	48±37	38 (19–66)	**0.001**	55±39	52 (28–75)	**0.001**
	Day 3	34±36	20 (10–45)		39±56	23 (12–42)	
	Day 5	32±37	12 (6–53)		32±69	15 (8–26)	
	Day 7	35±61	8 (3–30)		19.8±23.4	10.5 (4–27)	
Fibrinogen, mg/dL	Day 1	599±118	607 (504–675)	**0.001**	532±127	515 (464–610)	**0.001**
	Day 3	522±133	520 (415–607)		507±139	496 (405–563)	
	Day 5	493±128	475 (411–580)		496±137	470 (437–532)	
	Day 7	463±144	441 (376–523)		438±101	428 (361–529)	

IQR: interquartile range; AST: aspartate transaminase; LDH: lactate dehydrogenase; CRP: C-reactive protein. Bold indicates statistically significant p-value.

Oxygen status on days 3 and 7 after anti-inflammatory therapy was classified and compared according to the WHO clinical scale. Changes in the oxygen status according to the WHO clinical scale on days 3 and 7 between the high-dose steroid and anakinra+steroid groups were similar (p=0.976). Complications developed in 26 of 133 patients. Bacterial pneumonia was observed in 10 patients, bacteremia in 4 patients, ARDS in 9 patients, gastrointestinal system bleeding in two patients, and psychiatric problems in 3 patients. Complication rates were 26% in the anakinra+steroid group and 12% in the steroid group (p=0.03).

In the 28-day follow-up of the patients, 57% of the patients who died were detected in the anakinra+steroid group and 42% were in the steroid group, and no significant difference was observed between the 28-day mortality rates (p=0.48). Univariate regression analysis for affecting 28-day mortality, including age, sex, severity of CT findings, LDH, ALT, CRP, and ferritin levels, duration of steroid administration, and addition of anakinra, was performed (Table 3). Age, severe CT findings, and duration of steroid administration were found to be the factors affecting 28-day mortality. Multivariate regression analysis of anakinra addition was performed with factors affecting mortality, such as age, severity of CT findings, and LDH level. As a result of the regression analysis performed with these confounding factors, it was found that the addition of anakinra did not affect 28-day mortality (p=0.67).

**Table 3:** Univariate and multivariate analysis of parameters predicted to be effective on mortality.

Parameters	Univariate analysis			Multivariate analysis		
	OR	CI	p	OR	CI	p
Receiving anakinra	0.714	0.263–1.876	0.48	0.765	0.223–2.631	0.67
Age	1.052	1010–1.086	**0.02**	1.044	1.010–1.086	**0.03**
Ferritin, ng/mL	1.000	0.999–1.000	0.57			
ALT, IU/L	0.995	0.645–1.010	0.50			
CRP, mg/L	1.000	0.645–1.010	0.50			
LDH, IU/L	1.010	1.000–1.010	0.09			
Duration of steroid	1.123	1.103–1.237	**0.02**			
Severity of CT finding	1.754	0.344–2.941	**0.03**	3.100	1.058–9.300	**0.03**
Sex	0.970	0.357–2.631	0.94			

OR: odds ratio; CI: confidence interval; ALT: alanine transaminase; CRP: C-reactive protein; LDH: lactate dehydrogenase; CT: computed tomography. Bold indicates statistically significant p-value.

## DISCUSSION

In our study, the addition of anakinra to steroid treatment resulted in a significant decrease in the ferritin level in the first 7 days in patients. The ferritin and LDH values of the patients, which were checked at the time of admission to the hospital and on the day of MAS evaluation, were higher in the anakinra+steroid group, and there may be a bias against anakinra. Nevertheless, laboratory values were followed up for 7 days after anti-inflammatory treatment, and a significant decrease in ferritin levels was detected in the anakinra+steroid group (p=0.001). The effect of anakinra on ferritin decrease is consistent with the literature^5,6^. In a prospective cohort study of 60 critically ill COVID-19 patients followed up in the intensive care unit, ferritin reduction was more pronounced in the anakinra group. In the study by Emma et al., it was shown that the hyperinflammatory state regressed more rapidly in patients who were given anakinra^7^. Anakinra more effectively decreases the ferritin levels by inhibiting cytokine release and the inflammatory cycle with IL-1 blockade^4^. LDH reduction was found to be significant in both groups. In the literature, it is emphasized that high LDH levels may be associated with more severe disease course, hospitalization, and the need for intensive care^8^. LDH decrease was observed in the anakinra+steroid (p=0.001) and steroid (p=0.005) groups.

In our study, anakinra was administered intravenously, with a mortality rate of 16%. In the study by Huet et al., 100 mg of anakinra was administered subcutaneously twice a day in patients with saturation <93% and who did not need intensive care, and the mortality rate was found to be 25% at the 20-day follow-up. Also, steroid was not given to every patient in the control group^9^. In our study, mortality in the anakinra+steroid group was lower than that in other studies. This may be due to intravenous administration of anakinra. With the intravenous use of anakinra, there was no absorption problem, and the effective dose was reached quickly^10^. In our study, the mortality rate in the steroid group was 12%. In the study by Batirel et al., conducted with 189 patients, 250 mg methylprednisolone treatment for 3 days was compared with 6 mg dexamethasone treatment. In their study, while mortality was 5% in the pulse steroid arm, it was 11% in the 6 mg dexamethasone arm^11^. The reason for low mortality in their study was that it was accepted in mild patients and nondeveloping MAS. In our study, adding anakinra to treatment did not affect 28-day mortality (p=0.67). In a systematic review in which the efficacy of anakinra was evaluated in hospitalized patients with COVID-19, mortality did not decrease compared to placebo and standard treatment groups in patients receiving anakinra^12^. In another study, clinical outcomes were not better in patients receiving anakinra than the standard care group and placebo group^13^.

Our study had some limitations. First, it was conducted in a single center, which may limit the generalizability of the findings. Second, the anakinra+steroid group had the worst oxygen status before treatment. This could be attributed to the fact that anakinra was administered to patients with higher ferritin levels and poorer clinical conditions, potentially introducing selection bias. Additionally, further studies are required to investigate the effects of anakinra in more severe patient groups who require mechanical ventilation.

However, this study had several strengths. First, regular follow-ups of routine tests, IL-6, CK, and procalcitonin were performed regularly. Oxygen status was observed regularly for 7 days. However, data were collected retrospectively. Second, only the critical patient group, before going to the intensive care unit, was discussed.

## CONCLUSION

COVID-19 progresses to MAS with proinflammatory cytokine increase. In our study, the addition of anakinra to steroid treatment resulted in a significant decrease in ferritin and LDH levels in the first 7 days in patients who developed cytokine storm. However, multivariate analysis of the addition of anakinra found no effect on 28-day mortality. There was no significant difference in the magnitude of improvement in the oxygen status between the two treatment groups, according to the WHO's clinical scale. Clinicians should be aware of the complications of anti-inflammatory therapies. However, more controlled studies are needed to determine the effectiveness of high-dose steroid administration with anakinra.
